# Supplementary figures and images for: Anthropogenic Disturbance Impacts Gut Microbiome Homeostasis in a Malagasy Primate
Source: Front Microbiol. 2022 Jun 21;13:911275. doi: 10.3389/fmicb.2022.911275 (PMC9253676; doi:10.3389/fmicb.2022.911275)

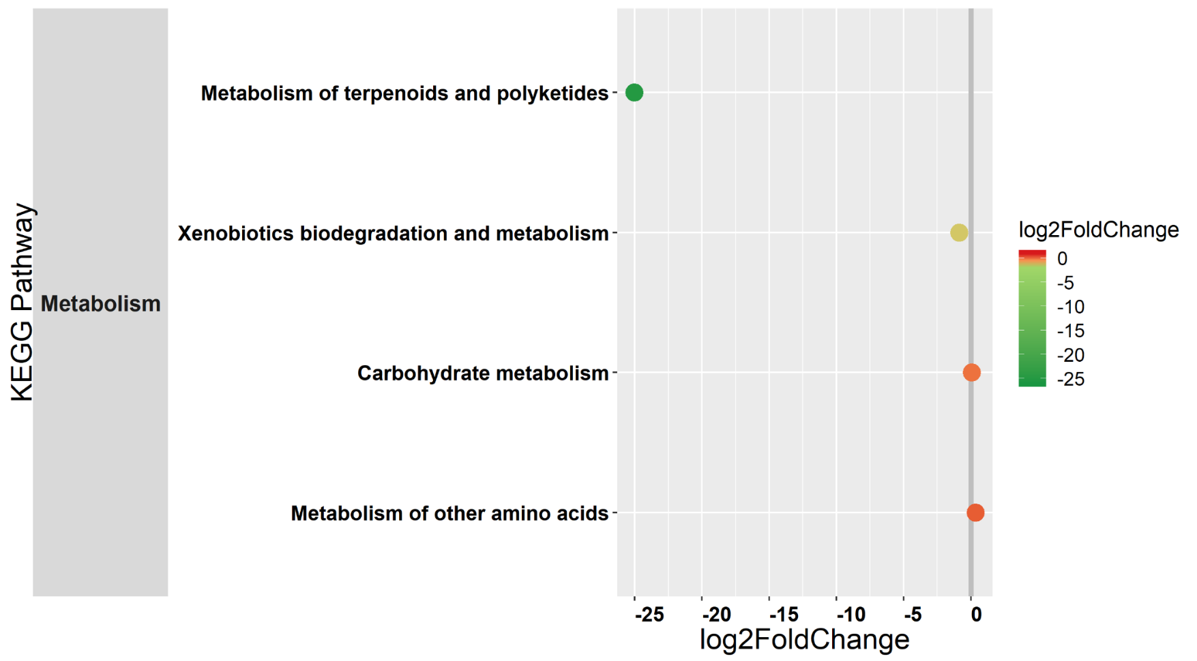

Supplement: Supplementary Figure 1 — Differential abundance of predicted major functional pathways in relation to the habitat of mouse lemurs. Differences in the mean abundance of major functional pathways (by using KEGG classification) between Andranovao and Miarintsoa individuals (Wald tests, p ≤ 0.05). The values indicate a log 2-fold decrease or increase in Miarintsoa individuals. Functional pathways are arranged according to increasing values of log 2-fold change. [file Image_1.TIF]
